# No need to beat around the bushmeat–The role of wildlife trade and conservation initiatives in the emergence of zoonotic diseases

**DOI:** 10.1016/j.heliyon.2021.e07692

**Published:** 2021-07-30

**Authors:** M.H. Hilderink, I.I. de Winter

**Affiliations:** Department of Biology, Utrecht University, Hugo R. Kruytgebouw, Padualaan 8, 3584 CH Utrecht, the Netherlands

**Keywords:** Emerging infectious disease, Zoonoses, Wildlife trade, Conservation, COVID-19, Biodiversity

## Abstract

Wildlife species constitute a vast and uncharted reservoir of zoonotic pathogens that can pose a severe threat to global human health. Zoonoses have become increasingly impactful over the past decades, and the expanding trade in wildlife is unarguably among the most significant risk factors for their emergence. Despite several warnings from the academic community about the spillover risks associated with wildlife trade, the ongoing COVID-19 pandemic underlines that current policies on the wildlife industry are deficient. Conservation initiatives, rather than practices that attempt to eradicate zoonotic pathogens or the wild species that harbour them, could play a vital role in preventing the emergence of life-threatening zoonoses. This review explores how wildlife conservation initiatives could effectively reduce the risk of new zoonotic diseases emerging from the wildlife trade by integrating existing literature on zoonotic diseases and risk factors associated with wildlife trade. Conservation should mainly aim at reducing human-wildlife interactions in the wildlife trade by protecting wildlife habitats and providing local communities with alternative protein sources. In addition, conservation should focus on regulating the legal wildlife trade and education about disease transfer and safer hunting and butchering methods. By uniting efforts for wildlife protection and universal concern for preventing zoonotic epidemics, conservation initiatives have the potential to safeguard both biodiversity, animal welfare, and global human health security.

## Highlights

• Wildlife trade results in the emergence of zoonoses.
• Tackling the wildlife trade can safeguard human health, biodiversity, and animal welfare.
• Conservation initiatives can play a vital role in preventing zoonotic disease emergence.
• Reducing the human-wildlife interface in the wildlife trade is a top priority for conservation.
• Regulating and monitoring legal wildlife trade and providing alternative proteins and education can aid in lowering zoonotic disease transmissions.

## 1 Introduction

On December 31, 2019, the Wuhan Municipal Health Commission informed the World Health Organisation (WHO) about nearly 40 cases of acute respiratory distress syndrome with unfamiliar aetiology in Wuhan City, China [1]. Deep sequencing analyses of lower respiratory tract samples quickly indicated a novel coronavirus emergence [2, 3]. The virus was called SARS-CoV-2 and the disease COVID-19, a name that would go down in history as the biggest threat to humanity since the Second World War [4]. Over the last months, the COVID-19 pandemic has spread with alarming speed, leading to the infection of millions of people and a demobilised global economy [4, 5]. Medical experts revealed that SARS-CoV-2 proved 96% similar to coronaviruses in bats, reinforcing its suspected zoonotic origin [6]. The virus possibly spilled over to humans through an unknown intermediate host at the Huanan Seafood Wholesale market in Wuhan, where wildlife species, including birds, snakes, frogs, marmots, wolf cubs, turtles, pangolins, civet cats, and bats, are sold illegally [1, 7]. As the COVID-19 health crisis continues to unfold globally, the potential threat of zoonotic pathogens emerging from wildlife has never been more apparent [8].

Wildlife species constitute a large and unknown reservoir for emerging infectious diseases that can pose severe threats to global human health and biodiversity [9]. Nearly 75% of all emerging infectious diseases are zoonotic, meaning that pathogens are transmitted from animals (usually vertebrates) to humans [10, 11], and the majority of zoonotic diseases (71.8%) originates from wild animals [10, 12]. Zoonoses have had substantial impacts on human and environmental health, as well as agricultural production and trade over the last decades [13, 14, 15]. For this reason, zoonoses originating from wildlife have received increased scientific attention, especially after the Severe Acute Respiratory Syndrome (SARS) pandemic in 2003 [16,17]. Yet, despite several warnings from the academic community about zoonotic spillover risks from wildlife, the management of zoonotic outbreaks is usually reactive and based on the vaccine and drug development after pathogen emergence, instead of focussing on spillover prevention [18, 19, 20]. The COVID-19 pandemic proves that such policies remain deficient.

The increase of zoonotic spillover from the wildlife reservoir has been linked to a sharp and exponential rise in global human activity, including human population expansion and encroachment of wildlife habitat, increasing land-use change and deforestation, growing demand for animal consumption-based food systems, and globalisation of travel and trade [14, 21, 22, 23, 24, 25, 26, 27]. A specific form of globalising trade is the wildlife trade, i.e., sourcing, selling, translocation, and consumption of wildlife commodities, which has expanded dramatically over recent years and is considered a major driver of population declines in mammals [16, 28]. Not only is wildlife trade severely damaging to biodiversity and animal welfare [29, 30, 31], the wildlife industry, comprised of activities that relate to the farming, trading, hunting, and breeding of wildlife, and all the services, products, as well as ecotourism, particularly increases contact between humans and wildlife, ultimately increasing zoonotic spillover risks [32, 33]. In addition, several aspects of the wildlife trade, like hunting and butchering [19, 34], bushmeat markets [35], and the import of live animals [36, 37], provide exceptional opportunities for successful pathogen transmission from wildlife to humans.

Adopting conservation approaches could provide a promising solution to prevent future zoonotic spillover from wildlife trade [38]. Conservation practices directly enhance the protection of biodiversity and animal welfare and would contribute to global human health security [39]. Especially now, with all eyes on the COVID-19 pandemic, conservation initiatives could impact the protection of wildlife while safeguarding human health and harmonising human-wildlife interactions. Thus, in this field, conservation objectives and global interest for preventing zoonotic epidemics could go hand in hand. This review explores how wildlife conservation initiatives could effectively reduce the risk of new zoonotic diseases emerging from the wildlife trade by integrating existing literature on zoonotic diseases and risk factors associated with wildlife trade. The cited literature was gathered from four online databases (Scopus, ResearchGate, Google Scholar, and Microsoft Academic Search) from January 1990 to June 2021, using search terms relating to the themes of *wildlife trade*, *zoonoses*, *emerging infectious diseases*, and *conservation*. We included papers in this review that reported on either the impacts of wildlife trade on emerging zoonotic diseases or the effects of conservation efforts on wildlife trade and/or zoonotic diseases. Although the wildlife trade also includes ranch-raised and captive-bred ‘wild’ animals, we focus on the trade of wild-caught animals. This review first analyses the zoonotic spillover risk factors of wildlife trade, divided into four trade phases, and hereafter explores the deficiencies of current zoonotic disease prevention strategies and the opportunities for implementing conservation methods to reduce zoonotic spillover risks.

## 2 Zoonotic spillover risk factors associated with wildlife trade

The wildlife trade is a multimillion-dollar business embedded in complex cultural contexts [40, 41, 42]. We distinguish between legal and illegal wildlife trade, given that illegal wildlife trade, i.e., the unlawful unsustainable harvesting of and trade in wildlife commodities, specifically targets endangered species due to their rarity and economic value, threatening to drive such species toward extinction. Estimates of 40,000 primates, 4 million birds, 640,000 reptiles, and 350 million exotic fish traded annually illustrate the enormous scale and impact of the wildlife industry altogether [13]. Wildlife trade brings wild animals in close proximity to humans and provides an interface for zoonotic spillover [33, 35, 43]. Wildlife along the trade route is exposed to hunters, marketers, and consumers, as well as to other domesticated and wild animal species that can potentially serve as vectors [44, 45]. This exposure leads to an estimate of one billion contacts between wildlife and humans annually that provide opportunities for zoonotic spillover [13]. Here, we discuss the risk factors of wildlife trade according to trade activities, divided into four phases. These phases are 1) the hunting, trapping, and butchering of wildlife, 2) the transportation of live animals and wildlife products, 3) the sale of wildlife at bushmeat markets and live animal markets, and 4) the consumption and use of bushmeat, wildlife products, and live animals.

### 2.1 Phase 1 – hunting, trapping, and butchering

The wildlife trade begins with the hunting of wild species, ranging from informal subsistence hunting to highly organised transnational trafficking for commercial purposes [41, 46]. Although subsistence hunting still exists, there are increasing trends of hunting villages selling wildlife for cash income, turning it into commercial wildlife trade [47]. Hunting practices have advanced tremendously over the past decades due to logging infrastructures and modern hunting equipment [48]. Nevertheless, the risk of zoonotic spillover during hunting and trapping intensifies with higher hunting rates. First, when hunters penetrate wildlife habitat, a considerable risk of indirect transmission through the environment arises [49, 50]. Various zoonotic pathogens, such as the monkeypox virus and other simian retroviruses, can spread indirectly through deposited body fluids and contaminated soils, waters, and other materials [51, 52]. Second, hunting and capturing animals involves direct physical contact, and humans are at risk of injuries, such as bites and scratches and, thereby, the exchange of bodily fluids that accompany such injuries [41, 53, 54]. Also, the butchering of wildlife poses a high risk for bloodborne and other bodily fluid transmissions of zoonotic pathogens [19]. During skinning, opening the body cavity, removing the organs, and cutting the meat, butchers may obtain injuries, like cuts from knives and bone fragments, and are in contact with the animal's blood and other body fluids [34, 53, 54]. Outbreaks of monkeypox and Ebola, as well as the emergence of retroviruses, like HIV, are linked to the butchering of wildlife, nonhuman primates specifically [52, 53, 55].

### 2.2 Phase 2 – transportation

Transportation of wildlife ranges from local barter to major international routes [13]. Such modern supply chains, which transport wildlife products over hundreds of kilometres, amplify the potential spread of zoonotic pathogens [16, 44]. Especially for pathogens that do not transmit between humans, the international transport of wildlife plays a crucial role in the global spread of zoonotic pathogens [19].

Trade of live and non-live wildlife are markedly different and pose specific risks for zoonotic spillover [56, 57]. The risks of importing non-live wildlife materials are increasingly recognised, as traders often neglect adequate preservation or cleaning procedures, certainly in the illegal trade [48, 58, 59]. In addition, several pathogens, like those of African swine fever and anthrax, are resistant to environmental changes and can cause foodborne transmission after importing raw or undercooked meat [58, 60]. The majority (92% of shipments in the US) of traded wildlife, however, concerns live animals for the trade in exotic pets, live market animals, zoo animals, and laboratory specimens [18]. This live animal trade poses risks of 1) cross-species spillover and 2) the release of vectors. First, during pre-export housing and transportation, unnaturally grouped animals are kept at high densities so that pathogens that are typically confined within their host reservoir can make the jump to other species, amplify, and possibly spill over to humans [36, 44]. Second, many traded animals escape or are intentionally released by their owners, potentially spreading pathogens to endemic species and other vectors [16, 48]. Moreover, vectors like exotic mosquitos and fleas may be accidently imported with animals, possibly spreading to endemic species or facilitating zoonotic spillover at their destination [61]. Finally, as animal health certificates are not required in many countries and illegally traded animals enter the country in passenger baggage, imported wildlife often has an unknown health status, possibly carrying novel zoonotic pathogens and posing a high risk for public health [36, 37, 60, 61, 62].

### 2.3 Phase 3 – sale

The sale of wildlife products to consumers, certainly of illegal products, is often conducted through informal networks [63]. However, the sale of wildlife products through commercial bushmeat markets and live animal markets is prevalent, and this type of sale is documented at least to some extent [35, 64, 65]. Several zoonotic outbreaks have been linked to the sale of wildlife products at markets. Avian influenza and SARS, for example, are related to the live trade of birds and carnivores on traditional wet markets, respectively [66, 67, 68]. Again, cross-species spillover and accidental release of vectors at bushmeat and live animal markets could explain such disease emergence [69]. A mixture of genera from different taxonomic classes is often present on wildlife markets, suggesting the presence of high pathogen diversity [35].

Another risk associated with markets is the live butchering of animals, which provides opportunities for bloodborne and other bodily fluid transmissions between humans and animals of different species [19, 54, 70]. Unhygienic conditions can also result in the presence of wild and domestic scavengers that consume the remnants and wastes from traded wildlife, potentially spreading wildlife pathogens or serving as vectors [13]. Additionally, the release of animals that have passed through markets contributes to the introduction and amplification of novel pathogens and vectors into the wild, increasing the probability of eventual spillover [13, 45]. Furthermore, the daily introduction of new individuals and day-to-day carryover at permanent markets support excellent conditions for pathogen amplification [65, 70]. Finally, keeping wild animals in stressful, unhygienic situations lowers their immune response and increases opportunities for cross-species pathogen transmission and amplification [31, 65, 71, 72].

### 2.4 Phase 4 – consumption and use

The final phase of the wildlife trade is the consumption and use of wildlife products. Although humans have consumed wildlife products and kept exotic animals as pets for millennia, the demand for both non-live products and live animals has increased dramatically over recent years [73, 74]. Central Africa and the Amazon basin, where 67–164 million kilograms of bushmeat, respectively, are consumed annually, illustrate the sheer magnitude of the growing demand for bushmeat [13, 41]. Besides the fact that demand for wildlife products drives the risky wildlife trade activities discussed above, the consumption of bushmeat, the use of wildlife products, and the keeping of wild animals present several risks for zoonotic disease emergence [69].

Many African hunting communities prefer bushmeat over domestic meat, as bushmeat consumption has strong cultural ties [75, 76]. Nevertheless, the emergence of multiple zoonotic diseases has demonstrated the global health risks related to the consumption of bushmeat [52, 69, 77, 78]. Fresh bushmeat, especially that of nonhuman primates, can transmit zoonotic pathogens like retroviruses and hepatitis [32, 52, 79]. For instance, the origin of HIV and severe Ebola outbreaks are linked to the consumption of nonhuman primates [53, 77, 78, 80].

The use of other wildlife products concerns traditional medicine, apparel, ornamentals, and biomedical and pharmaceutical industries. Though pathogens are less likely to survive on material surfaces than bushmeat, several zoonotic spillover cases through wildlife products exist [48, 58]. Examples are the outbreak of anthrax after the use of goat hides for making drums, as well as sparganosis infections resulting from the use of traditional medicine made from frog skin or snake bile [58, 59]. Still, the main risk for zoonotic spillover due to wildlife product use is the demand for these products that drives hunting, butchering, transport, and sales activities [41, 81].

Finally, keeping exotic animals as pets or zoo animals is associated with several zoonoses, varying from parasitic ringworm infections to severe monkeypox and lyssaviruses, associated with prairie dogs and pet bats, respectively [32]. Private exotic pet collections and zoological collections raise various wild animals in high-density, unnatural groupings, increasing cross-species transmission and eventual spillover risks. Exotic pets and zoo animals, if infected, can bring zoonotic pathogens into close contact with the animal caretakers, and inadequate care or scratch and bite injuries can facilitate zoonotic pathogen transmission [61].

Concluding, activities along the wildlife trade and supply chain, ranging from hunting to consumption, pose a high risk for zoonotic pathogen transmission and disease emergence. Given that those zoonotic pathogens spread through various transmission pathways, sometimes multiple pathways at the same time, e.g., through (in)direct physical contact, bodily fluids, and faecal-oral, foodborne, and airborne transmission, a single trade activity can have a drastic impact on the spread and amplification of zoonoses (Table 1). The practices and places within these four phases have not been studied to such an extent that causal relations between specific practices and zoonoses can be established [82]. Links are found between environmental factors, including landscape and climatic factors, and disease emergence, but these relationships vary with the species, pathogens, and circumstances involved [83]. Nevertheless, it is clear that wildlife trade affects human health security, biodiversity, and animal welfare, and thus tackling the wildlife trade can safeguard all of these components at once [84].

**Table 1:** Risk factors associated with the wildlife trade.

Trade phase	Trade activity	Risk factor	Transmission pathway
1)	Hunting and trapping	Penetrating wildlife habitat Injuries during capture (bites, scratches, other)	Indirect physical contact Direct physical contact Body fluids
	Butchering	Contact with animal's skin, organs, blood, etc. Injuries during butchering (knives, bone fragments)	Direct physical contact Body fluids (specifically bloodborne) Body fluids (specifically bloodborne)
2)	Transport of non-live wildlife products	Raw bushmeat, inadequate preservation and cleaning of bushmeat and other products	Indirect physical contact Foodborne Faecal-oral
	Translocation of live animals	High-density unnatural groupings of wildlife species (Accidental) translocation and release of vectors	Cross-species pathogen spread and amplification Vector-borne
	Legal wildlife transport	Lack of regulation, pathogen surveillance, and enforcement	(In)direct physical contact Body fluids Foodborne Faecal-oral Airborne Vector-borne
	Illegal wildlife transport	Lack of wildlife transport regulations, unknown health status of animals	(In)direct physical contact Body fluids Foodborne Faecal-oral Airborne Vector-borne
3)	Sale of wildlife on bushmeat markets and live animal markets	High-density unnatural groupings of wildlife species, daily carryover (Accidental) release of vectors Unhygienic conditions, presence of scavengers Live butchering	Cross-species pathogen spread and amplification Vector-borne (In)direct physical contact Body fluids Foodborne Faecal-oral Airborne Vector-borne Body fluids (specifically bloodborne)
4)	Consumption of bushmeat	Driver of hunting, butchering, transport, and sale Touching of infected bushmeat Consumption of infected bushmeat	(In)direct physical contact Body fluids Foodborne Faecal-oral Airborne Vector-borne Direct physical contact Foodborne
	Use of wildlife products (medicine, ornamentals, apparel, other materials)	Driver of hunting, butchering, transport, and sale Use of traditional medicine containing processed wildlife products Use/wear of other wildlife products	(In)direct physical contact Body fluids Foodborne Faecal-oral Airborne Vector-borne Direct physical contact Foodborne Faecal-oral Direct physical contact Faecal-oral
	Keeping live wild animals	Driver of hunting, transport and sale Close contact between wild animal and caretaker, risk of injuries (scratches/bites) Unnatural groupings of wildlife species	(In)direct physical contact Body fluids Foodborne Faecal-oral Airborne Vector-borne (In) direct physical contact Body fluids Faecal-oral Airborne Cross-species pathogen spread and amplification

This table summarises zoonotic spillover risk factors associated with the wildlife trade. These risk factors are grouped according to wildlife trade phases 1–4 and specific trade activities within this phase. Phase 1: hunting, trapping, and butchering. Phase 2: transportation. Phase 3: sale. Phase 4: consumption and use. The risk factor indicates what particular behaviour/condition concerning the trade activity poses a zoonotic spillover risk. Hereafter, the zoonotic pathogen's transmission pathway is shown, i.e., what transmission pathways do the risk factors promote.

## 3 Zoonoses and wildlife conservation

### 3.1 The current state of conservation strategies against the wildlife trade

The wildlife trade is strongly linked to the extinction of wild species [29, 30, 73, 85]. Up to 50% of traded species on live animal markets are endangered, and the observed endangered species are thought to present only a fraction of the total traded endangered wildlife [35, 48]. For decades, both small and large conservation organisations have made extensive efforts to halt the wildlife industry. Initiatives range from raising public awareness and educating local community members, to supporting governments in regulating wildlife trade and training law enforcement officers, to carrying out foot patrols, removing traps and snares, and developing poacher detection techniques [86, 87, 88, 89]. Furthermore, organisations collect data on wildlife trafficking, monitor wildlife markets, and carry out undercover investigations to expose illegal markets and smugglers.

However, the effectiveness of various conservation initiatives is under debate. For example, illegalising the wildlife trade has pushed it further underground, making it even harder to monitor [36, 65, 90]. In addition, the risks (e.g., punishments, fines, or imprisonment) for illegal wildlife trade are relatively low compared to the benefits and are minimal relative to other illegal industries [91]. Moreover, international agreements like the Convention of Biological Diversity and the CITES convention (i.e., the Convention on International Trade in Endangered Species of Wild Fauna and Flora), which aim to ensure that international trade in wild species does not threaten their survival, also have undesirable effects on the wildlife trade. For instance, trade in non-CITES listed species, therefore labelled as ‘legal’ wildlife trade, often has an unrecognised illegal background, i.e., legally binding rules are broken with impunity. This ‘legal’ wildlife trade remains widely accepted, as it carries no stigma and is not punishable by law [92]. Also, international trade regulations, like the EU Wildlife Trade Regulations, prescribe various spillover prevention procedures for the legal wildlife trade (e.g., import permits, animal health certificates, hygiene standards, pre-export quarantine, microbial control), but in practice, effective enforcement and adequate monitoring techniques are lacking, so that zoonotic disease emergence risks persist [61, 92]. While many conservation organisations focus on the illegal wildlife trade, the legal wildlife trade is an expanding, problematic industry that poses severe threats of zoonotic pandemics [48, 93].

Before the outbreak of SARS-CoV-2, wildlife trade conservation projects mainly concentrated on ending the illegal wildlife industry in light of biodiversity conservation and animal welfare, while a focus on preventing zoonotic epidemics was lacking [88, 94, 95]. Though the potential of wildlife conservation practices to combat zoonoses emerging from the wildlife industry is now increasingly recognised, this potential is rarely used when implementing zoonotic outbreak prevention strategies [8, 96, 97, 98]. Moreover, with a lack of focus on zoonotic spillover prevention and governmental policies depending on reactive vaccine development after zoonotic disease emergence [19], zoonoses will progressively emerge at higher frequencies and magnitudes [11, 12]. Reoccurring zoonotic disease outbreaks, like those of Severe Acute Respiratory Syndrome (SARS) in 2003, Middle East Respiratory Syndrome (MERS) in 2012, and finally COVID-19 in 2019, underline the continuing effects of wildlife trade, consumption, and disturbances of natural human-wildlife interactions on human health security and remind us that the current management of zoonotic disease outbreaks remains inadequate [99].

### 3.2 Conservation strategies for zoonotic spillover prevention

Rising numbers of emerging infectious diseases originating from wildlife demonstrate the need for alternative, effective zoonotic spillover prevention strategies [8, 11, 12, 100, 101]. We believe that conservation organisations could play a critical role in mitigating spillover risks from the wildlife trade, as well as in improving animal welfare, and could use global health security as a unique selling point to gain international attention and support for their practices. Here, we discuss the potential of adopting conservation strategies that benefit biodiversity protection and reduce zoonotic disease emergence risks from the wildlife trade. We outline the implementation of three conservation methods for spillover prevention and point out prevention approaches and conservation dilemmas that require further investigation.

#### 3.2.1 Reducing the human-wildlife interface

Decreasing contact rates between humans and wildlife, rather than attempting to eradicate pathogens or the wild species that harbour them, is among the most crucial steps in reducing zoonotic spillover risks [13, 38, 101, 102]. Deforestation and land-use changes, which increase human-wildlife contact rates, are strongly linked to the emergence of zoonotic diseases [8, 26, 54, 103, 104, 105, 106, 107]. The protection of wildlife habitats, which is currently among the main goals of many conservation organisations, can counter forest loss and mitigate the human-wildlife interface and should, therefore, be a top priority for conservation [16, 32, 102, 108]. The strict protection of wildlife habitat, or ‘land sparing’, is necessary to avert habitat disturbances that increase contact rates between wildlife and humans. Especially the prevention of internal habitat disturbances, like the development of infrastructure, decreases human-wildlife contact, lowers hunting rates, and restrains the global spread of zoonoses that emerge in indigenous communities [19, 23, 100, 108, 109, 110]. Moreover, the protection of wildlife habitats slows down biodiversity loss [48, 109]. Although higher biodiversity houses a larger zoonotic pathogen pool, the ‘dilution effect’ model predicts that high species diversity results in decreased transmission of zoonotic diseases [45, 111, 112]. Also, high species diversity avoids the homogenisation of biota, which reduces pathogens' ability to move between different habitats and amplify across regions [18, 113, 114]. Thus, conservation organisations should identify areas with high biodiversity and high-risk species and protect these areas by strengthening protected area boundaries and buffer zones, implementing advanced anti-poaching techniques, and stimulating wildlife stewardship by local communities [26, 100, 115, 116, 117].

Whereas land sparing methods separate grounds for human use and conservation purposes, land sharing integrates biodiversity conservation and food production in the same area [118]. Land-sharing practices, also known as agroforestry, can provide significant economic benefits to locals. However, conflicts between wildlife and humans on shared grounds are common and, therefore, land sharing poses undesirable risks for zoonotic spillover [108, 119, 120]. Yet again, the fact that strictly protected areas are primarily of low economic value to local communities and governments, gaining support for preserving such areas is problematic, putting these protected areas under pressure and presenting a conservation dilemma that requires further investigation [119, 121]. Therefore, site-specific risk assessments for human-wildlife contacts and potential disease emergence are needed.

For reducing the human-wildlife interface, another conservation dilemma rears its head. Ecotourism practices are, on the one hand, a valid source of income for conservation organisations and local communities but, on the other hand, require the development of accommodations and infrastructure in wildlife habitats and bring tourists from all over the world near wildlife and zoonotic pathogens [32]. The mountain gorillas in Bwindi Impenetrable National Park, Uganda, which attract over 20,000 tourists each year, illustrate the massive impact of ecotourism on the human-wildlife interface and spillover risks [122]. Most eco-tourists, however, view wildlife at a distance and have no direct, physical contact with wildlife. Therefore, the potential for transmission between eco-tourists and wildlife is considered minimal relative to the bushmeat trade [123]. Also, the involvement of local communities as actors in gorilla tourism has helped to counter poaching and trophy hunting and has increased local and governmental support for preserving the forest instead of clearing it for agricultural usage [124]. Overall, site and species-specific risk assessments are needed on the relative spillover risks and financial and conservation benefits involved with eco-tourism [125].

#### 3.2.2 Regulation of the legal wildlife trade

Though different opinions exist on whether to ban all kinds of wildlife trade entirely or not, several studies suggest that further illegalising the wildlife trade may only increase underground (illicit) trade, eliminating the possibility of monitoring safe trading conditions [36, 48, 65, 84, 126, 127]. Besides, since millions of people depend on the legal wildlife trade to sustain their livelihoods and have no direct economic alternatives, completely banning the wildlife trade would be impossible [40, 65, 84, 100, 126]. As the impacts of wildlife trade bans possibly jeopardise transparent trading conditions and affect spillover risks, further research on this topic is required before such trade bans can in any way be implemented safely. Therefore, in the first place, instead of prohibiting the wildlife trade altogether, conservation organisations should support the well-regulated, safe, sustainable, and cruelty-free trade of wild species whilst continuing to combat the illegal, unsustainable, unhygienic, and abusive trade of wildlife [128]. To counter illegal wildlife trade, conservation organisations need to support governments and international bodies like the UN in the development of advanced anti-poaching and anti-trafficking techniques and efficient poacher/smuggler prosecution systems, the training of law enforcement officers, and the spread of awareness campaigns to reduce the demand for wildlife products and exotic pets [100, 126, 127, 128, 129, 130].

Although the role of wildlife in emerging zoonoses is well established, regulations on wildlife trade are often not enforced or non-existent and lack organisation and responsibility [48]. For instance, in the US, there is a little coordinated national strategy, legislative authority, or funding devoted to the oversight of wildlife trade [18]. Nevertheless, strict and well-adhered to trade regulations that apply to domesticated animals, especially in the EU, demonstrate that the regulation of legal wildlife trade is certainly not impossible [32, 61, 131]. Therefore, conservation organisations should actively engage in establishing and enforcing international guidelines for the harvesting, slaughter, transportation, and sale of wild animals, and develop procedures and certification for sustainable, commercial farming of wild animals with low disease transmission risk [13, 35, 75, 84, 100, 132, 133, 134]. Additionally, we believe that we should take responsibility for the welfare of captured wildlife and for mitigating unacceptable impacts, as animal welfare is often compromised in the wildlife trade, especially in the illegal, wild-caught trade of live animals [31]. For instance, after capture, live animals are often kept close to humans and other animals, including predators, and many animals die during transportation, for example, through crushing, starvation, dehydration, or stress, and never make it into trade [135, 136]. Hygiene, sanitary, and welfare standards, as provided by the World Organisation for Animal Health, contribute to improving animal stress levels and immune response, consequently decreasing their susceptibility to zoonotic pathogens [31, 72, 84, 100]. Moreover, hygiene and sanitary protocols, e.g., providing clean drinking water, adequate waste disposal and ventilation, and personal protective equipment, prevent direct spillover events during the handling and processing of wildlife meat and products [61, 84, 92, 100]. Finally, prohibiting unnatural, high-density groupings of wild (and domestic) species along the wildlife trade chain is critical to avoid cross-species spillover and amplification [36, 44, 84, 100].

An essential aspect of regulating the legal wildlife trade includes the surveillance of zoonotic pathogens in exported and imported animals [19, 42, 93]. For instance, the major outbreaks of the West Nile virus and monkeypox in North America, caused by single shipments of infected animals, could have been prevented by proper pathogen surveillance [32]. Even though there are many time-consuming challenges to develop and execute appropriate pathogen surveillance systems, effective pathogen monitoring in the domestic trade demonstrates the efficacy of these systems for avoiding zoonotic disease emergence [8, 19, 36, 48, 61]. Therefore, conservation organisations need to support the establishment of a more cost-effective, decentralised disease surveillance system for transported and farmed wildlife, educate and train workers to detect early signs of disease, and supervise the proper execution of proactive, routine wildlife pathogen screening [61, 84, 93, 100].

#### 3.2.3 Local community support, education, and involvement

Local communities are key to preventing wildlife trade activities and corresponding zoonotic disease emergence [137, 138]. Rural communities often depend on subsistence hunting or commercial wildlife trade to sustain their livelihoods and are, therefore, one of the primary sources behind the hunting and butchering of wildlife [41, 76, 96]. To reduce local hunting rates and the zoonotic spillover risks associated with hunting, conservation initiatives should support local communities in providing alternative sources of protein and income [41, 70, 139]. Notwithstanding, such conservation efforts might have ambiguous effects on bushmeat hunting, as economic development may paradoxically increase the demand for efficient hunting materials and bushmeat consumption [139, 140]. Hence, further exploring the provisioning of local alternative protein sources, specifically, and of auxiliary health-risk education to restrain increasing hunting rates, is essential to successfully reduce local hunting and mitigate spillover risks.

Local awareness of zoonotic diseases can also contribute to reducing bushmeat hunting rates. While conservation-education programmes are unlikely to impact local hunting and wildlife use due to the cultural and religious significance of bushmeat, health-risk education has the potential to minimise hunting rates of species, like nonhuman primates, that pose a particular risk to human health [53, 63, 70, 141]. Still, Friant et al. [76] found that individuals did protect themselves against infection despite being aware of zoonotic disease risks. So, as bushmeat hunting is likely to continue, education programmes should include methods to hunt and butcher more safely. Examples include immediately disinfecting injuries like bites, scratches, and cuts obtained during hunting or butchering and avoiding butchering or handling meat if one has wounds on his hands or arms [53]. Also, hygiene-health education, e.g., how to properly dispose of wastes and separate drinking water from water used for washing animal products, can aid in lowering spillover risks [84, 100]. Protective strategies should be developed using locally available goods and should be tested for cultural acceptability [76].

Finally, the importance of involving local communities in combating the illegal wildlife trade and corresponding spillover risks is increasingly recognised [46]. Based on their proximity to and knowledge of wildlife, communities are well suited to detect, report on, and help to prevent poaching and trafficking activities. Adequate governance and stewardship of rural communities' rights can strengthen local support for conservation efforts and increase the effectiveness of counter-poaching initiatives [46]. Policies on both the illegal and legal wildlife trade should not be made without the active and prior involvement of local communities [128, 142, 143].

### 3.3 Conservation practices of the future: focus on biodiversity and human health

Various conservation initiatives have the potential to reduce zoonotic spillover risks emerging from the wildlife trade. Conservation organisations embody expertise on (illegal) wildlife trade and are, therefore, well-placed to act on preventing zoonotic disease emergence [144]. However, the implementation and profitability of short- and long-term conservation actions should be given serious consideration. For instance, strictly denying human access to protected areas may reduce the human-wildlife interface and spillover risks, but at the same time throws up barriers to economic development and possibly violates local human rights as well as their local tradition and beliefs [100, 142, 145].

With the ongoing COVID-19 pandemic, a shift in focus from biodiversity protection to human health security is specifically convenient. The One Health approach, which aims to combat health issues like zoonoses at the human-animal-environment interface, is a helpful example of how conservation initiatives could integrate biodiversity protection and health security purposes [146, 147, 148]. To achieve such a transnational One Health approach, conservation organisations will primarily need to engage in cross-sectoral dialogue and activities to strengthen international policymaking, effective enforcement, the involvement of local communities, and cooperation between zoonotic disease and wildlife experts, international legislators, and other conservation organisations [13, 46, 133]. Moreover, creating awareness of zoonotic disease risks and biodiversity threats posed by the wildlife trade to impact the social acceptability and demand for wildlife products, e.g., through media campaigns and education, will remain one of conservation organisations' most significant responsibilities [16, 38, 86, 129].

## 4 Prospects and recommendations

The wildlife trade poses a high risk of zoonotic disease emergence and is severely damaging to biodiversity and animal welfare. Nevertheless, given the rising demand for wildlife products and the vast amount of people depending on the wildlife industry to sustain their livelihoods, the wildlife trade is unlikely to come to an end [40, 41]. If, though, wildlife trade continues like today or intensifies even further, an increasing trend in both the frequency and magnitude of zoonotic disease emergence can be expected [11, 12]. Especially viral zoonotic pathogens are predicted to pose a significant threat of zoonotic disease emergence in the future, which is well-illustrated by SARS-CoV-2 [11,[149], [150], [151]]. Under the influence of the growing human population, climate change, declining biodiversity, globalisation of transport, and expanding trade in wildlife, the increasing trend of emerging zoonotic diseases presumably persists [12, 16, 18, 45, 111, 112, 152].

Conservation organisations are well-positioned to address the international wildlife trade, prevent zoonotic disease emergence, and safeguard human health security [48]. The current outbreak of COVID-19 underscores the importance of zoonotic spillover prevention and has significantly increased international attention and support for acts to abolish and regulate the wildlife trade [153]. In summary, conservation initiatives should aim at reducing human-wildlife interactions by protecting wildlife habitat, regulating the legal wildlife trade, and supporting, educating, and involving local communities to combat the illegal wildlife trade. As conservation organisations cannot address disease emergence threats alone, a cross-sectoral disease prevention approach is essential. For this reason, conservation organisations will have to engage in and support, among others, international policymaking and the enforcement of disease prevention measures.

In conclusion, conservation organisations could wield a worldwide concern for emerging zoonotic diseases, sparked by the COVID-19 pandemic, to implement wildlife trade conservation measures that could potentially safeguard biodiversity, animal welfare, and global human health security simultaneously. Conservation initiatives that reduce zoonotic spillover risks are crucial in preventing the emergence of zoonotic diseases. Instead of trying to ‘flatten the curve’ of people infected with zoonoses once they have emerged, like with COVID-19, the time has come to flatten the curve of zoonotic disease emergence itself.

## Declarations

### Author contribution statement

All authors listed have significantly contributed to the development and the writing of this article.

### Funding statement

This research did not receive any specific grant from funding agencies in the public, commercial, or not-for-profit sectors.

### Data availability statement

Data included in article/supplementary material/referenced in article.

### Declaration of interests statement

The authors declare no conflict of interest.

### Additional information

No additional information is available for this paper.
